# Supplementary material for: The histone H3K9 demethylase KDM3A promotes anoikis by transcriptionally activating pro-apoptotic genes BNIP3 and BNIP3L
Source: eLife. 2016 Jul 29;5:e16844. doi: 10.7554/eLife.16844 (PMC4991936; doi:10.7554/eLife.16844)
Supplement: Figure 1—source data 1. — DOI: http://dx.doi.org/10.7554/eLife.16844.004 [file elife-16844-fig1-data1.docx]

| **Fold enrichment (detached vs. attached)** | **shRNA sequence** | **Gene symbol** | **Gene name** |
| --- | --- | --- | --- |
| 1620.792946 | TAACACTTCACTGTAACTC | *ZCCHC24* | zinc finger CCHC-type containing 24 |
| 1324.931564 | AAATGCTTCACAATCAAAG | *KDM3A* | lysine demethylase 3A |
| 1139.789798 | TAATCTTCAAATAAGCTGA | *RALBP1* | ralA binding protein 1 |
| 947.6848454 | TCACTTACAAGTTTCTTTC | *RFC1* | Replication factor C subunit 1 |
| 848.4223889 | AATATAAGGATTGCTATCG | *LRIG3* | leucine-rich repeats and immunoglobulin like domains 3 |
| 798.0503961 | TTAAGTAGCTTAGAGAGGG | *PIH1D3* | PIH1 domain containing 3 |
| 720.0231915 | TGTCATTCCAAGAGATCCT | *DKK1* | dickkopf WNT signaling pathway inhibitor 1 |
| 710.1463302 | ATTTGCCGGAGGATCAAGG | *SENP6* | SUMO1/sentrin specific peptidase 6 |
| 681.0095893 | AATCTAATACATTAATCTG | *PLEKHG1* | pleckstrin homology and RhoGEF domain containing G1 |
| 671.1327279 | TTGGAGATGAGGCTCAGTG | *FGD5* | FYVE, RhoGEF and PH domain containing 5 |
| 665.2066111 | TAATCCAATAATTCATTTC | *LOC339524* |  |
| 659.2804943 | TTAAAATGGAGACACCATC | *METAP1D* | methionyl aminopeptidase type 1D (mitochondrial) |
| 645.3541198 | ATTCCTTCAAGATTTCAAG | *VMP1* | vacuole membrane protein 1 |
| 633.6006548 | TCTCTGATGCTGAGTAAGG | *ZNF345* | zinc finger protein 345 |
| 619.2792059 | TTCTATCGAATAAGCAATC | *FAM227B* | family with sequence similarity 227 member B |
| 610.5022036 | TTACGTTTCTGAATTTCTG | *PDE7A (variant 2)* | phosphodiesterase 7A |
| 609.6640814 | AACTTATAGACATTCAGAC | *GPC5* | glypican 5 |
| 605.9454431 | TTCCCTCACATGTGGGATG | *AP4M1* | adaptor related protein complex 4 mu 1 subunit |
| 598.4192748 | ATTGAAATATTGAAACTTC | *SUSD1* | sushi domain containing 1 |
| 561.9934101 | ATAGAGAAAGTCTTTATAC | *RBFOX3* | RNA binding protein, fox-1 homolog (C. elegans) 3 |
| 559.5241948 | TAGAATAAGAACTACTGTC | *DNAJB9* | DnaJ heat shock protein family (Hsp40) member B9 |
| 543.6619555 | TTAATCTTATCTTTGCCTG | *CYP39A1* | cytochrome P450 family 39 subfamily A member 1 |
| 527.4243955 | TGAAAGACTTAACAATTGG | *CECR5-AS1* | CECR5 antisense RNA 1 |
| 526.4367093 | TAACTTCAAAGGTGTATCC | *ADH7* | alcohol dehydrogenase 7 (class IV), mu or sigma polypeptide |
| 518.5352203 | ATAGATATATGCATTTAGG | *RNF125* | ring finger protein 125, E3 ubiquitin protein ligase |
| 513.1029465 | TATTAGGAATCTTAACCAC | *HOXA11* | homeobox A11 |
