# Supplementary material for: The histone H3K9 demethylase KDM3A promotes anoikis by transcriptionally activating pro-apoptotic genes BNIP3 and BNIP3L
Source: eLife. 2016 Jul 29;5:e16844. doi: 10.7554/eLife.16844 (PMC4991936; doi:10.7554/eLife.16844)
Supplement: Supplementary file 1. — DOI: http://dx.doi.org/10.7554/eLife.16844.032 [file elife-16844-supp1.docx]

**Supplementary file 1.** List of shRNAs obtained from Open Biosystems/Thermo Scientific.

| **Gene** | **shRNA ID** |
| --- | --- |
| *BIM* | TRCN0000001051 |
| *BIM-2* | TRCN0000001052 |
| *BNIP3* | TRCN0000007830 |
| *BNIP3-2* | TRCN0000007832 |
| *BNIP3L* | TRCN0000007844 |
| *BNIP3L-2* | TRCN0000007847 |
| *KDM3A* | TRCN0000021152 |
| *KDM3A-2* | TRCN0000021151 |
| *Kdm3a* (mouse) | V3LHS_394642 |
| *Kdm3a-2* (mouse) | V2LMM_150878 |
| *METAP1D* | TRCN0000052160 |
| *METAP1D-2* | TRCN0000052161 |
| *PIH1D3* | V3LHS_319344 |
| *PIH1D3-2* | V3LHS_319345 |
| *ZCCHC24* | TRCN0000148931 |
| *ZCCHC24-2* | TRCN0000149482 |
| *ZNF345* | TRCN0000016728 |
| *ZNF345-2* | TRCN0000016729 |
