# Supplementary material for: The histone H3K9 demethylase KDM3A promotes anoikis by transcriptionally activating pro-apoptotic genes BNIP3 and BNIP3L
Source: eLife. 2016 Jul 29;5:e16844. doi: 10.7554/eLife.16844 (PMC4991936; doi:10.7554/eLife.16844)
Supplement: Supplementary file 2. — DOI: http://dx.doi.org/10.7554/eLife.16844.033 [file elife-16844-supp2.docx]

**Supplementary file 2.** List of primers used for qRT-PCR and ChIP.

| **Gene** | **Forward primer sequence (5’ 🡪 3’)** | **Reverse primer sequence (5’ 🡪 3’)** | |  |
| --- | --- | --- | --- | --- |
| **qRT-PCR** | | | |  |
| *BAD* | AAGACTCCAGCTCTGCAGAG | | ATGATGGCTGCTGCTGGTTG |  |
| *BAK1* | TCGACTTCATGCTGCATCAC | | ACAAACTGGCCCAACAGAAC |  |
| *BAX* | ACTGGACAGTAACATGGAGCTG | | AAACATGTCAGCTGCCACTC |  |
| *BBC3* | ACCTCAACGCACAGTACGAG | | ACCTAATTGGGCTCCATCTCG |  |
| *BID* | TGTTCTGACAACAGCTTCCG | | ATCAGTCTGCAGCTCATCGTAG |  |
| *BIK* | TGGCTTTCATCTACGACCAGAC | | GTGGTGAAACCGTCCATGAAAC |  |
| *BIM* | TGCAGACATTTTGCTTGTTCAA | | GAACCGCTGGCTGCATAATAAT |  |
| *BMF* | GAGGTACAGATTGCCCGAAA | | TTCAAAGCAAGGTTGTGCAG |  |
| *BNIP3* | ACGAGCGTCATGAAGAAAGG | | AATCCGATGGCCAGCAAATG |  |
| *BNIP3L* | TGCGAGGAAAATGAGCAGTC | | TGCCATTGCTGCTGTTCATG |  |
| *BOK* | ACATCTCCCTGCAGTCTGAG | | TGCCTGCAGAGAAGATGTGG |  |
| *Gapdh* | TGCACCACCAACTGCTTAGC | | GGCATGGACTGTGGTCATGAG |  |
| *HRK* | CACCAGCGCACCATGTGG | | CAGCCAAGGCCAGTAGGTG |  |
| *KDM3A* | GAGTTCAAGGCTGGGCTATTGT | | TTCAGCCACTTTGATGCAGCTA |  |
| *Kdm3a (mouse)* | ATTCGAGCTGTTTCCCACAC | | TCCAAGACTCCCCATCAAAC |  |
| *METAP1D* | AACAACGTGCTCTGTCATGG | | ACCACATTCGTCCACATTGC |  |
| *HUWE1* | TCACCGCACTGTGTTAAACC | | TGCGCTTGACATCAAAGTCG |  |
| *PMA1P1* | AGAGCTGGAAGTCGAGTGTG | | GAAGTTTCTGCCGGAAGTTCAG |  |
| *PIH1D3* | TATTCAGACAGCAGGTGGGAAC | | AGCCACTAGTTCACTGCAAC |  |
| *RPL41* | CATTAAATAGCCGTAGACGGAACTT | | GCGCAGAGGTTTCCAAAAAA |  |
| *ZCCHC24* | TGCCACATCAACGTGTATCCA | | GGCCGTCGGGCTTCTC |  |
| *ZNF345* | AAACAGGGATCTCAGGAAGGAC | | GGATACTGAAAGTGGGCATGTC |  |
| **ChIP** | | | |  |
| *BNIP3* | CACTAGCAGGATGGAAAGACG | | ACTCTCTGGGCACTGGCTAC | |
| *BNIP3L* | AGATACCCTGGGTAGCAGTAAC | | ACCCGGTAAAGAACTAGCAGAG | |
| *KDM3A* | CGCTTGTAAAATGGGAGGCATG | | TTGTGTGCTCTGGACCTGAAG | |
| *NCR (GCLC)* | ATGGTTGCCACTGGGGATCT | | TGCCAAAGCCTAGGGGAAGA | |
